# BAG3 Attenuates Ischemia-Induced Skeletal Muscle Necroptosis in Diabetic Experimental Peripheral Artery Disease

**DOI:** 10.3390/ijms231810715

**Published:** 2022-09-14

**Authors:** Arul M. Mani, Karthik Dhanabalan, Victor Lamin, Thomas Wong, Madhu V. Singh, Ayotunde O. Dokun

**Affiliations:** 1 Division of Endocrinology and Metabolism, Carver College of Medicine, University of Iowa, Iowa City, IA 52242, USA; 2 Fraternal Order of Eagles Diabetes Research Centre, Carver College of Medicine, University of Iowa, Iowa City, IA 52242, USA

**Keywords:** BAG3, peripheral artery disease, diabetes, autophagy, necroptosis

## Abstract

Peripheral artery disease (PAD) is characterized by impaired blood flow to the lower extremities, resulting in ischemic limb injuries. Individuals with diabetes and PAD typically have more severe ischemic limb injuries and limb amputations, but the mechanisms involved are poorly understood. Previously, we identified BAG3 as a gene within a mouse genetic locus termed limb salvage QTL1 on mouse chromosome 7 that determined the extent of limb necrosis following ischemic injury in C57Bl/6 mice. Whether BAG3 deficiency plays a role in the severe ischemic injury observed in diabetic PAD is not known. In vitro, we found simulated ischemia enhanced BAG3 expression in primary human skeletal muscle cells, whereas BAG3 knockdown increased necroptosis markers and decreased cell viability. In vivo, ischemic skeletal muscles from hind limbs of high-fat diet (HFD)-fed mice showed poor BAG3 expression compared to normal chow diet (NCD)-fed mice, and this was associated with increased limb amputations. BAG3 overexpression in ischemic skeletal muscles from hind limbs of HFD mice rescued limb amputation and improved autophagy, necroptosis, skeletal muscle function and regeneration. Therefore, BAG3 deficiency in ischemic skeletal muscles contributes to the severity of ischemic limb injury in diabetic PAD, likely through autophagy and necroptosis pathways.

## 1. Introduction

Peripheral artery disease (PAD) is a form of atherosclerotic occlusive disease outside of the heart that commonly affects the lower extremities [1]. It has been estimated that worldwide, over 200 million people have PAD [1,2]. Individuals with PAD typically present with claudication, which is pain with ambulation, or chronic limb-threatening ischemia (CLTI), which involves ischemic rest pain or ischemic ulcerations [1,2]. Diabetes mellitus (DM) is one of the major risk factors for developing PAD [3,4,5,6]. When DM is present with PAD, the disease is more likely to involve arteries distal to the knee, and affected individuals are five times more likely to have disease requiring an amputation than non-diabetic patients with PAD [7,8]. Moreover, of the risk factors for developing CLTI, diabetes has the most significant impact [3,4,5,6]. Therefore, ischemic limb injury is more severe in diabetes. The molecular mechanisms of diabetes that contribute to the more severe ischemic injury in diabetes are poorly understood, but it has been hypothesized that DM can modify PAD outcomes by increasing the progression of atherosclerosis in lower extremity vessels [6,8,9,10,11]. We and others have hypothesized that diabetes can modify post-ischemic adaptive mechanisms involved in restoring blood flow and tissue repair following vessel occlusion [6,8,9,10,11,12,13].

In ischemic limb injuries, a major determinant of the severity of tissue damage is the survival of skeletal muscle cells in ischemia [14,15,16,17]. Additionally, the extent of skeletal muscle injury, cell survival and the likelihood of amputation in response to ischemia is influenced by the genetic background [18,19,20,21] of individuals and mice. We previously identified a genetic locus termed limb salvage QTL1 (LSQ-1) on mouse chromosome 7 that determined the extent of limb necrosis following ischemic injury in a mouse model of PAD (hind limb ischemia or HLI) [21]. We have since identified that the BCL2-associated athanogene 3 (BAG3) gene, one of the genes within LSQ-1, contributes to the extent of skeletal muscle injury and adaptation following experimental PAD [22,23].

BAG3 is a 575 amino acid member of the BAG family of proteins. It is expressed in all mammalian tissue but most prominently in the heart and skeletal muscles [24]. Interestingly, BAG3-deficient mice developed a fulminant myopathy [25]. In humans, defects in BAG3 have been linked to severe childhood muscular dystrophy [26], and there is evidence that BAG3 may regulate autophagy in certain cell types [27,28,29,30,31]. We previously showed that a variant of BAG3 expressed in C57BL/6 mice was protective against ischemic injury [22]. Nevertheless, our knowledge about the role of BAG3 in the regulation of ischemic skeletal muscle injury is limited, and whether BAG3 plays a role in the more severe ischemic limb injury associated with diabetes is not known.

Skeletal muscle death following injury may occur through different mechanisms [32,33]. Recent studies suggest myofiber death in dystrophin-deficient mice may occur through necroptosis [34]. There is also evidence that defective autophagy may enhance necroptosis [35]. Whether ischemia induces necroptotic cell death in skeletal muscle cells is not known. Additionally, whether autophagy and necroptosis pathways play a major role in the severity of skeletal muscle pathology in diabetic PAD is not known.

The aim of the present study is to investigate a mechanism contributing to the increased likelihood of developing a more severe ischemic injury in diabetic PAD using a mouse model of PAD with diabetes. We hypothesize that levels of BAG3 expression play a role in the severity of skeletal muscle injury following experimental diabetic PAD. Additionally, we hypothesize that ischemic skeletal muscles in diabetic PAD undergo death by necroptosis, and this can be regulated by BAG3 through modulation of autophagy.

## 2. Results

### 2.1. Ischemia Enhances BAG3 Expression in Primary Human Skeletal Muscle Cells (HSMC)

**Figure 1:**
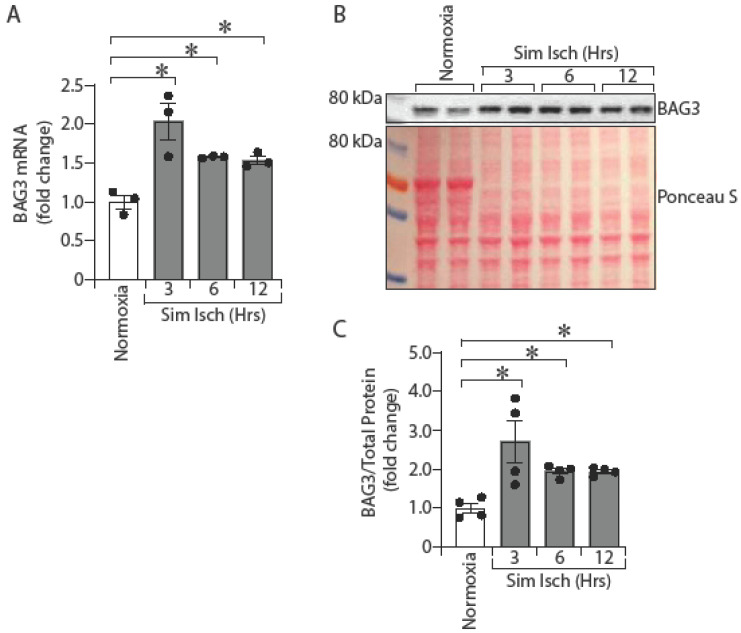
Ischemia enhances BAG3 mRNA and protein expression in HSMCs. HSMCs were exposed to simulated ischemia (Sim Isch), and cell lysates were collected at 0, 3, 6, and 12 h. BAG3 mRNA was determined by TaqMan qPCR (**A**) *n* = 3 and BAG3 protein was measured by Western blot analysis (**B**) *n* = 4. The protein band was quantified and normalized to total protein from Ponceau S staining (**C**) *n* = 4. The results are presented as fold changes vs. normoxia. All the values are mean ± SEM. * *p* < 0.05 vs. normoxia.

Skeletal muscle cells constitute the bulk of the limb muscle tissue. We therefore investigated the expression of BAG3 in primary muscle cells in vitro. To better understand the role of BAG3 in ischemic skeletal muscle, we determined whether BAG3 is induced in human skeletal muscle cells under ischemic stress. BAG3 mRNA expression was significantly increased in cultured human skeletal muscle cells (HSMC) at 3 h after exposure to simulated ischemia and remained elevated during the 12 h of in vitro study (Figure 1A; non-ischemic, 1.0 ± 0.09; 3 h simulated ischemia, 2.03 ± 0.23; 6 h simulated ischemia, 1.58 ± 0.01; 12 h simulated ischemia, 1.53 ± 0.05, * *p* < 0.05). Similar to the mRNA results, BAG3 protein expression was also significantly increased at 3 h after exposure to simulated ischemia and remained elevated during the experiment when compared to cells grown under non-ischemic conditions (Figure 1B,C; non-ischemic, 1.0 ± 0.13; 3 h simulated ischemia, 2.71 ± 0.55; 6 h simulated ischemia, 1.95 ± 0.07; 12 h simulated ischemia, 1.94 ± 0.05, * *p* < 0.05). Thus, BAG3 expression is induced in skeletal muscle cells upon ischemic exposure.

### 2.2. Mice with Diabetes Have Decreased BAG3 Expression in Ischemic Limbs

**Figure 2:**
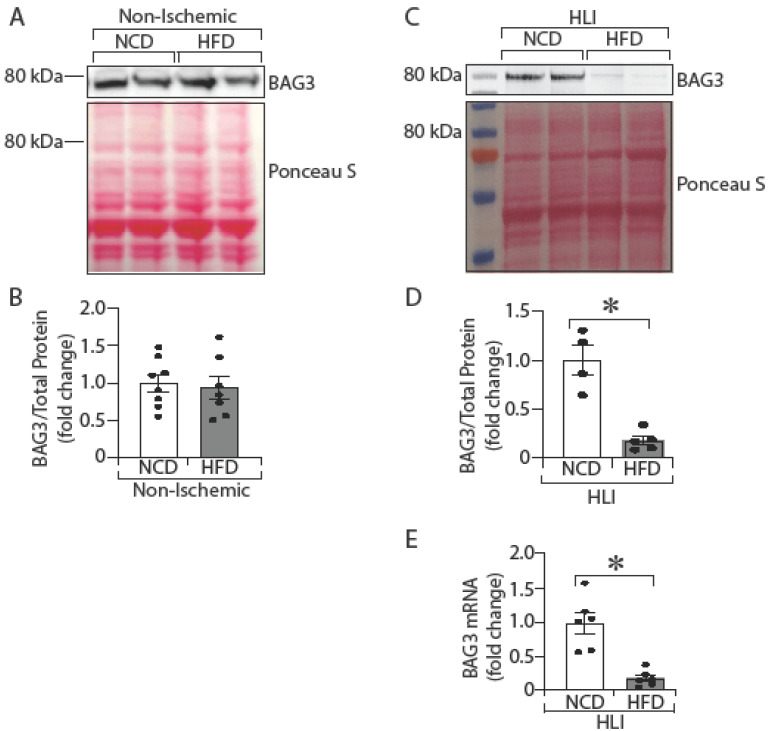
Mice with diabetes have less BAG3 expression in ischemic muscle. C57BL/6J mice were fed with normal chow diet (NCD) or high-fat diet (HFD) for 16 weeks, and hind limb ischemia (HLI) was induced on left limb of mice. After 3 days of HLI, gastrocnemius (GA) muscle samples were collected from both left (Ischemic) and right (Non-Isc; non-ischemic) limbs. BAG3 protein (**A**,**B**) *n* = 7–8 was quantified in non-ischemic GA muscles by Western blotting and was normalized with total protein from Ponceau S staining. BAG3 protein (**C**,**D**) *n* = 4–5 was quantified in ischemic GA muscle by Western blotting and was normalized with total protein from Ponceau S staining. Additionally, BAG3 mRNA (**E**) *n* = 6 was determined in ischemic GA muscle by TaqMan qPCR. The results are presented as fold changes vs. NCD. All the values are mean ± SEM. * *p* < 0.05 vs. NCD.

Humans with diabetes are more likely to develop CLTI, a form of PAD associated with severe ischemic limb injury. They also have a very high risk of limb amputation [36,37]. Given our prior studies that showed a variant of BAG3 was protective against ischemic limb injury, we hypothesized that the levels of BAG3 expression in ischemic skeletal muscles in diabetics may play a role in the severity of ischemic injury in diabetic PAD. We, therefore, explored the level of BAG3 expression in non-ischemic and ischemic skeletal muscles from non-diabetic normal chow diet (NCD)-fed and diabetic high-fat diet (HFD)-fed mice. In the non-ischemic GA muscles, we found no difference in BAG3 protein expression in the NCD-fed mice compared to the HFD-fed mice (Figure 2A,B; NCD vs. HFD, 1.0 ± 0.12 vs. 0.94 ± 0.15; *p*-value = 0.75). In contrast, ischemic HFD mice GA muscles expressed less BAG3 protein (Figure 2C,D; NCD vs. HFD, 1.0 ± 0.15 vs. 0.18 ± 0.04; * *p* < 0.05) and mRNA (Figure 2E; NCD vs. HFD, 1.0 ± 0.15 vs. 0.17 ± 0.04; * *p* < 0.05) than control GAs from NCD fed mice. Thus, in non-ischemic skeletal muscles, BAG3 expression is similar between non-diabetic NCD-fed mice and diabetic HFD-fed mice. However, in ischemic skeletal muscles, BAG3 mRNA and protein expression are significantly reduced in the HFD-fed mice compared to levels in the NCD-fed mice. These results suggest poor BAG3 expression in ischemic skeletal muscles in diabetes may play a role in the severity of limb injury in diabetic PAD.

### 2.3. In Vitro, BAG3 Knockdown Decreases Cell Viability, Reduces Autophagy and Enhances Necroptosis in HSMCs

**Figure 3:**
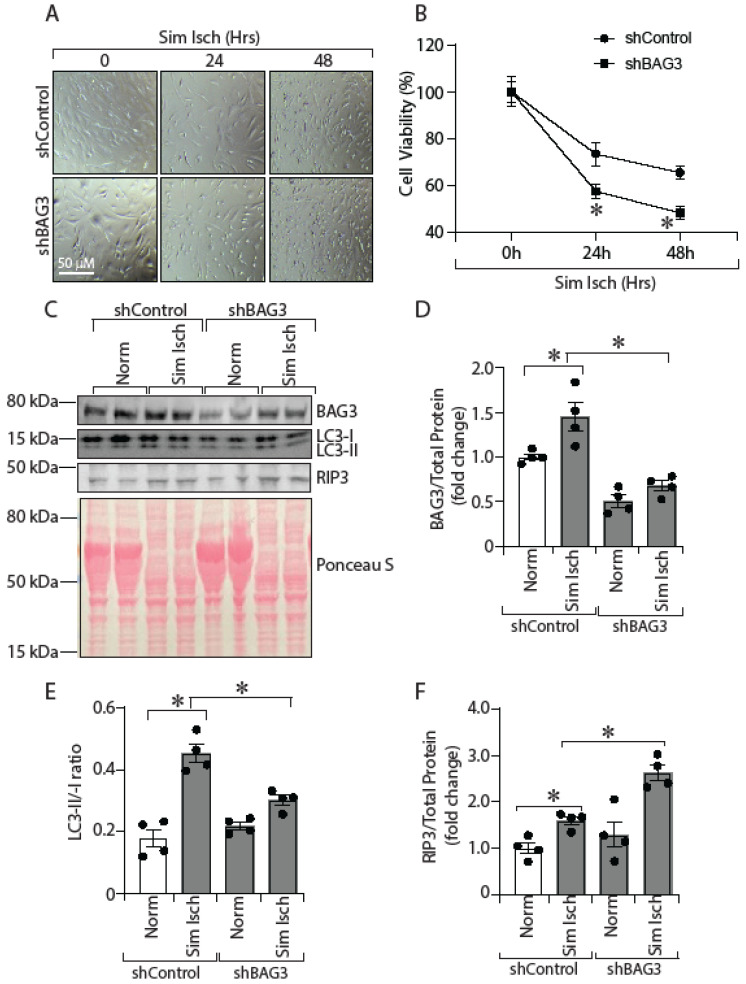
BAG3 knockdown decreases cell viability, reduces autophagy and enhances necroptosis in HSMCs. HSMCs transfected with shControl or shBAG3 were exposed to simulated ischemia for 0, 24, and 48 h and were micrographed at each indicated period (**A**). At the end of the indicated time, cells were stained with trypan blue, and viable cells were counted with a hemocytometer using an inverted microscope. The results are presented as percentage (%) cell viability compared to non-ischemic cells (**B**) *n* = 3. In addition, BAG3-depleted HSMCs cells were exposed to simulated ischemia for 6 h. Cell lysates were collected, and BAG3 (**C**,**D**) *n* = 4, LC3-II/-I ratio (**C**,**E**) *n* = 4 and RIP3 (**C**,**F**) *n* = 4 were analysed by Western blotting. The protein band was quantified and normalized to total protein from Ponceau S staining. The results are presented as fold changes vs. shControl-Norm. All the values are mean ± SEM. * *p* < 0.05 vs. shControl-Norm. Norm = normoxia; Sim Isch = simulated ischemia.

To better understand the role of reduced BAG3 expression on the severity of skeletal muscle injury in ischemia, we knocked down BAG3 in HSMCs and exposed the cells to ischemia, followed by an assessment of cell viability. We knocked down BAG3 in HSMCs by shRNA transfection (shBAG3) and subjected cells to simulated ischemia. As expected, simulated ischemia resulted in a time-dependent decrease in cell viability of HSMCs; however, cells with BAG3 protein knockdown showed lower cell viability than control shRNA-treated cells (Figure 3A,B; shControl vs. shBAG3, 0 h, 100% ± 3.7 vs. 100% ± 2.54; 24 h, 73.44% ± 2.9 vs. 57.5% ± 1.65; 48 h, 65.48% ± 1.7 vs. 48.2% ± 1.59, * *p* < 0.05). These results suggest BAG3 plays a role in skeletal muscle viability following ischemic stress. To investigate the possible mechanisms involved, we assessed the possible role of autophagy and necroptosis. Skeletal muscle cell death has been shown to occur through necroptosis, and defective autophagy has been implicated in enhancing necroptosis [35]. Additionally, BAG3 has been implicated in the regulation of autophagy [22,38]. Therefore, we knocked down BAG3 in HSMCs, exposed the cells to simulated ischemia and measured the expression of autophagy (LC3) and necroptosis (RIP3) markers. Transfection of BAG3-specific shRNA resulted in significantly decreased BAG3 protein in HSMCs (Figure 3C,D; shControl vs. shBAG3, 1.00 ± 0.03 vs. 0.50 ± 0.06, * *p* < 0.05). Exposure of BAG3-depleted HSMCs to 6 h of simulated ischemia was associated with decreased LC3-II/-I ratio, suggesting decreased autophagy (Figure 3C,E; LC3-II/-I ratio in shControl vs. shBAG3, 0.45 ± 0.03 vs. 0.30 ± 0.01, * *p* < 0.05). In addition, cells with BAG3 knockdown also had increased expression of RIP3, suggesting increased necroptosis (Figure 3C,F; RIP3 protein, shControl vs. shBAG3, 1.60 ± 0.09 vs. 2.63 ± 0.16, * *p* < 0.05). Together, these results suggest lower BAG3 expression in ischemic skeletal muscle cells is associated with decreased cell survival, likely through decreased autophagy and increased necroptosis.

### 2.4. In Vivo, Ischemic Skeletal Muscle in Diabetic Mice Shows Decreased Autophagy and Increased Necroptosis

**Figure 4:**
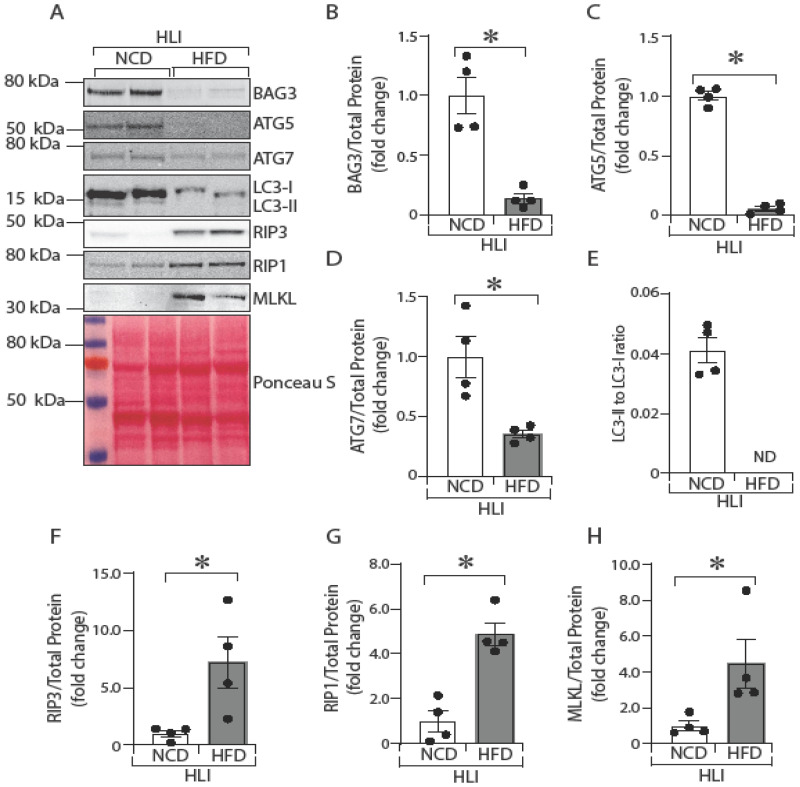
Ischemic skeletal muscle in diabetic mice shows decreased autophagy and increased necroptosis. C57BL/6J mice were fed with HFD for 16 weeks, and HLI was performed. After 3 days of HLI, GA muscle samples were collected, protein lysates were prepared, and BAG3 (**A**,**B**) *n* = 4, ATG5 (**A**,**C**) *n* = 4, ATG7 (**A**,**D**) *n* = 4, LC3-II/-I ratio (**A**,**E**) *n* = 4, RIP3 (**A**,**F**) *n* = 4, RIP1 (**A**,**G**) *n* = 4, and MLKL (**A**,**H**) *n* = 4 were quantified by Western blotting. The protein band was quantified and normalized to total protein from Ponceau S staining. The results are presented as fold changes vs. NCD. ND = not determined (because of undetectable band intensity). All the values are mean ± SEM. * *p* < 0.05 vs. NCD.

Next, we investigated whether the lower BAG3 protein expression in ischemic GA muscles of diabetic mice is associated with decreased autophagy and increased necroptosis. We compared the expression of BAG3 and autophagic and necroptosis markers in ischemic GA muscles from NCD-fed non-diabetic mice to HFD-fed diabetic mice that were subjected to experimental PAD (hind limb ischemia, HLI). Western blot analyses of GA muscle lysates from mouse hind limbs 3 days after HLI showed lower expression of BAG3 protein in the diabetic mice (Figure 4A,B; NCD vs. HFD, 1.0 ± 0.16 vs. 0.14 ± 0.03, * *p* < 0.05). Lower BAG3 expression was associated with lower expression of autophagy markers ATG5 (Figure 4A,C; NCD vs. HFD, 1.0 ± 0.03 vs. 0.06 ± 0.02, * *p* < 0.05), ATG7 (Figure 4A,D; NCD vs. HFD, 0.99 ± 0.17 vs. 0.35 ± 0.03, * *p* < 0.05), and undetectable band intensity of LC3-II (Figure 4A,E; NCD vs. HFD, 0.04 ± 0.004 vs. not detected) in ischemic GA muscle of mice with diabetes compared to non-diabetic controls. In contrast, there was higher expression of the necroptosis markers RIP3 (Figure 4A,F; NCD vs. HFD, 0.99 ± 0.27 vs. 7.23 ± 2.2, * *p* < 0.05), RIP1 (Figure 4A,G; NCD vs. HFD, 0.99 ± 0.47 vs. 4.88 ± 0.51, * *p* < 0.05), and MLKL (Figure 4A,H; NCD vs. HFD, 1.00 ± 0.27 vs. 4.48 ± 1.37). These results show that the lower BAG3 expression in ischemic GA muscles of diabetic mice (Figure 1) is associated with decreased autophagy and increased necroptosis.

### 2.5. BAG3 Overexpression Improves Autophagy and Decreases Necroptosis in Ischemic GA Muscles in Diabetes

**Figure 5:**
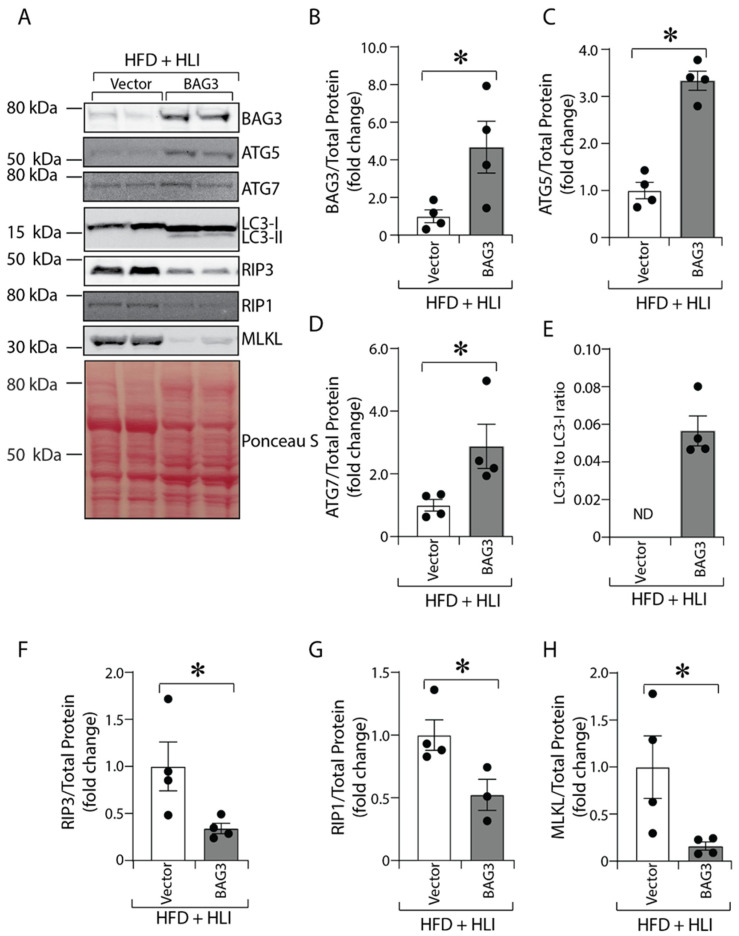
BAG3 overexpression improves autophagy and decreases necroptosis in ischemic skeletal muscles in diabetes. C57BL/6J mice were fed with HFD for 16 weeks. Mice were treated with AAV6-CMV (Vector) or AAV6-BAG3 (BAG3). After 7 days of HLI, GA muscle samples were collected, protein lysates were prepared, and BAG3 (**A**,**B**) *n* = 4, ATG5 (**A**,**C**) *n* = 4, ATG7 (**A**,**D**) *n* = 4, LC3-II/-I ratio (**A**,**E**) *n* = 4, RIP3 (**A**,**F**) *n* = 4, RIP1 (**A**,**G**) *n* = 3–4, and MLKL (**A**,**H**) *n* = 4 were quantified by Western blotting. The protein band was quantified and normalized to total protein from Ponceau S staining. The results are presented as fold changes vs. control vector. ND = not determined (because of undetectable band intensity). All the values are mean ± SEM. * *p* < 0.05 vs. Vector.

Our in vitro and in vivo results showed that lower BAG3 expression under ischemic stress is associated with decreased skeletal muscle cell viability, increased necroptosis and decreased autophagy. Hence, we hypothesized that replenishing BAG3 would im-prove autophagy and reduce necroptosis in ischemic skeletal muscles in mice with diabetes. To test this, we injected either control AAV6-EV (empty vector) or AAV6-BAG3 (vector with BAG3 cDNA) in the GA and TA muscles of HFD-fed diabetic mice prior to HLI surgery. Ischemic GA muscles of diabetic mice treated with BAG3 show increased expression of BAG3 protein on day 7 after HLI (Figure 5A,B; HFD + Vector vs. HFD + BAG3, 1.00 ± 0.34 vs. 4.67 ± 1.3, * *p* < 0.05). Consistent with our hypothesis, the increase in BAG3 protein resulted in an increase in the expression of autophagy markers ATG5 (Figure 5A,C; HFD + Vector vs. HFD + BAG3, 1.0 ± 0.17 vs. 3.33 ± 0.20, * *p* < 0.05), ATG7 (Figure 5A,D; HFD + Vector vs. HFD + BAG3, 1.0 ± 0.19 vs. 2.87 ± 0.70, * *p* < 0.05) and LC3-II to LC3-I processing (Figure 5A,E; HFD + Vector vs. HFD + BAG3; not detected vs. 0.056 ± 0.008). Moreover, this was associated with a decrease in the expression of necroptosis markers RIP3 (Figure 5A,F; HFD + Vector vs. HFD + BAG3, 1.00 ± 0.26 vs. 0.29 ± 0.03, * *p* < 0.05), RIP1 (Figure 5A,G; HFD + Vector vs. HFD + BAG3, 1.00 ± 0.12 vs. 0.52 ± 0.11, * *p* < 0.05), MLKL (Figure 5A,H; HFD + Vector vs. HFD + BAG3, 1.0 ± 0.33 vs. 0.16 ± 0.04, * *p* < 0.05). These results high-lighted an important role of BAG3 in preventing tissue injury through enhanced autophagy and abated necroptosis in ischemic skeletal muscles of diabetic mice.

### 2.6. BAG3 Overexpression Improves Limb Necrosis Score, Perfusion Recovery, Muscle Function and Muscle Regeneration in Diabetic PAD

**Figure 6:**
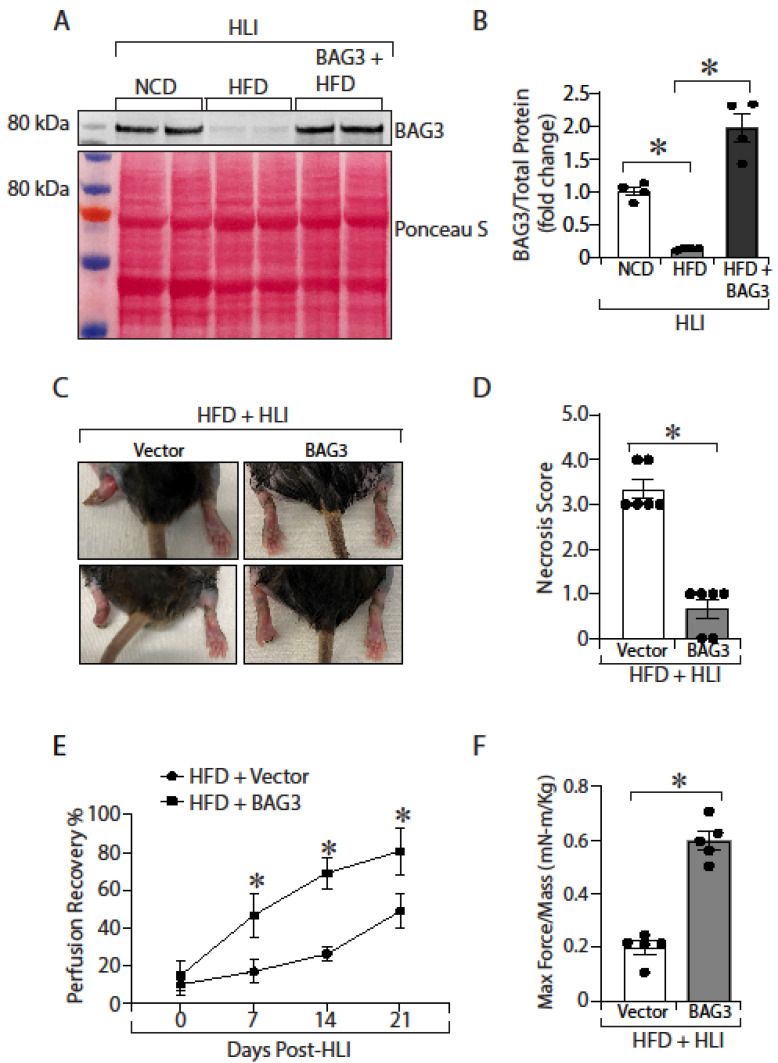

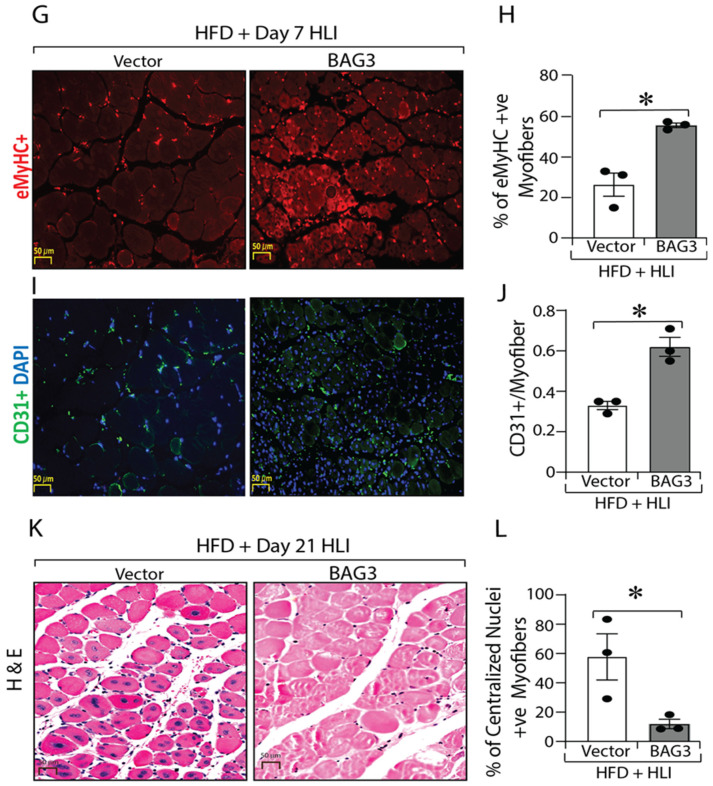
BAG3 overexpression improves necrosis score, perfusion recovery, muscle function and muscle regeneration in diabetic PAD. HLI surgery was performed on mice fed with HFD for 16 weeks. Mice were treated with AAV6-CMV (Vector) or AAV6-BAG3 (BAG3). BAG3 overexpression was confirmed in a subset of treated mice on day 7 post-HLI (**A**,**B**) *n* = 4. Necrosis score (**C**,**D**) *n* = 6, perfusion recovery (**E**) *n* = 4–6, and skeletal muscle function (**F**) *n* = 5 was determined. eMyHC+ staining (**G**,**H**) *n* = 3, CD31+ staining (**I**,**J**) *n* = 3 and H & E staining (**K**,**L**) *n* = 3 were carried out in GA muscle tissue. All the values are mean ± SEM. * *p* < 0.05 vs. vector.

We evaluated the physiologic effect of replenishing BAG3 in ischemic skeletal muscles of mice with diabetes. We measured limb necrosis score, perfusion recovery, and muscle function. As expected, AAV-mediated overexpression of BAG3 achieved higher levels of BAG3 protein in the ischemic GA muscles of treated diabetic mice. Further, we confirmed that BAG3 was overexpressed in ischemic GA muscle of HFD-fed mice by adenoviral injection (Figure 6A,B; NCD vs. HFD vs. HFD + BAG3, 1.0 ± 0.06 vs. 0.12 ± 0.01 vs. 1.97 ± 0.22). Overexpression of BAG3 significantly improved the necrosis score (see methods for scoring criteria) in treated diabetic limbs (Figure 6C,D; HFD + Vector vs. HFD + BAG3, 3.3 ± 0.21 vs. 0.67 ± 0.21, * *p* < 0.05). Evaluation of perfusion recovery revealed diabetic mice with BAG3 overexpression had significantly improved perfusion recovery compared to the control vector-treated diabetic mice (Figure 6E; Day 21 perfusion; HFD + Vector vs. HFD + BAG3, 46.02% ± 3.25 vs. 80.93% ± 6.4, * *p* < 0.05). Additionally, muscle function measurements of maximum muscle contraction in the hind limbs of the diabetic mice overexpressing BAG3 showed remarkable improvement in limb function when compared to diabetic mice treated with the control vector (Figure 6F; Day 21; HFD + Vector vs. HFD + BAG3, 0.19 ± 0.02 vs. 0.59 ± 0.03, * *p* < 0.05).

Moreover, examination of histological sections by immunohistochemistry revealed greater number of embryonic myosin heavy chain (eMyHC) positive muscle fibers in BAG3 overexpressing diabetic mice compared to the control vector treated diabetic mice (Figure 6G,H; Day 7; % of eMyHC+ fibers/total myofibers; HFD + Vector vs. HFD + BAG3; 26% ± 6.0 vs. 56% ± 1.1, * *p* < 0.05). Lastly, immunohistochemistry for CD31+ cells to quantify capillary density, a measure of angiogenesis in the ischemic limbs, showed higher capillary density (capillaries/myofiber) in the ischemic GA muscles of diabetic mice treated with BAG3 compared with those treated with control vector (Figure 6I,J; Day 7; capillaries/myofiber; HFD + Vector vs. HFD + BAG3, 0.33 ± 0.02 vs. 0.62 ± 0.05, * *p* < 0.05). In addition, histological examinations of GA muscles from Day 21 post-HLI showed that the percentage of muscle fibres with centralised nuclei was significantly reduced in the BAG3-treated GA muscles compared to the GA muscles from control vector-treated diabetic mice (Figure 6K,L; Day 21; % of centralized nuclei/total myofiber; HFD + Vector vs. HFD + BAG3, 58% ± 16.0 vs. 12% ± 3.0, * *p* < 0.05). Taken together, our results are consistent with BAG3 replenishing improving structural and functional ischemic damage to diabetic skeletal muscles by reducing limb necrosis, improving angiogenesis, and enhancing perfusion recovery and skeletal muscle regeneration.

## 3. Discussion

Chronic limb-threatening ischemia (CLTI) is a severe form of PAD associated with a high likelihood of limb amputation. Individuals with diabetes are five times more likely to develop CLTI [7,8]. Similar to the observation in humans in a preclinical model of PAD, mice with diabetes (HFD) develop a more severe ischemic injury following experimental PAD and are more likely to develop necrotic limbs and limb loss [3,4,5,6,7,8]. Although many studies have begun to explore the role of post-ischemic vascular adaption in poor PAD outcomes in diabetes, much less is known about the role of the skeletal muscle in poor PAD outcomes, such as that seen in CLTI [12,13]. Our previous efforts to understand the role of genetic backgrounds in the development of CLTI and poor post-ischemic vascular adaptation led to the identification of the Limb Salvage QTL-1 or LSQ-1 locus on mouse chromosome 7 [21]. We later identified BAG3 as a gene within LSQ-1 that was associated with protection from ischemic limb injury [22].

In this study, we hypothesized that the amount of BAG3 expression may play a role in the extent of ischemic skeletal muscle injury in diabetic mice following experimental PAD. In our in vitro experiments, BAG3 was induced in human skeletal muscle cells by simulated ischemia in a time-dependent manner, whereas depletion of BAG3 by shRNA resulted in a decreased number of live cells, suggesting a pro-survival role of BAG3. In our prior studies, we found increased autophagy flux in ischemic skeletal muscle of non-diabetic mice expressing a variant of BAG3 [22], suggesting BAG3 may regulate post-ischemic adaptation through modulation of autophagy. In this study, ischemic diabetic GAs express less BAG3 than ischemic non-diabetic GAs, and this was associated with less autophagy flux, while overexpression of BAG3 in ischemic diabetic GAs resulted in increased autophagy flux. These results further support the role for BAG3 in modulating autophagy in ischemic skeletal muscles. Skeletal muscle pathology in humans with CLTI and in preclinical models of PAD includes tissue fibrosis and evidence of cell death; however, the nature of skeletal muscle cell or myofiber death in humans with CLTI and in preclinical models of PAD is poorly understood. Recent evidence shows myofiber death in dystrophin-deficient mice occurs through necroptosis. Whether ischemia induces skeletal muscle cell death through necroptosis was not known. Our in vitro data showed increased necroptosis marker expression in human skeletal muscle cells exposed to simulated ischemia with BAG3 knocked down. Moreover, in our in vivo preclinical model of PAD with diabetes, ischemic diabetic GAs showed less BAG3 expression and higher expression of necroptosis markers, whereas overexpression of BAG3 in ischemic diabetic GAs resulted in lower expression of necroptosis markers. These results strongly suggest ischemia induces skeletal muscle cell death through necroptosis, and this is modulated by BAG3. In our studies, BAG3 modulates both autophagy and necroptosis, suggesting a link between the two processes. Our results are consistent with findings from prior studies by Lim et al. that showed autophagy plays a critical role in the turnover of RIP1 and RIP3 [35]. They further showed that defective autophagy resulted in increased necroptosis [35]. Taken together, these results are consistent with increased necroptosis in ischemic diabetic skeletal muscles due to reduced autophagy and BAG3 overexpression in the ischemic diabetic skeletal muscles decreasing necroptosis and improving autophagic flux.

To understand the physiologic consequence of BAG3 overexpression in ischemic limbs of mice with diabetes, we evaluated perfusion recovery, the extent of limb injury and limb function in these mice. We also performed a histological assessment of the ischemic GAs. Our results show that ectopic overexpression of BAG3 improved perfusion recovery, decreased limb necrosis and improved limb function in diabetic mice following induction of experimental PAD. AAV6-mediated overexpression of BAG3 in ischemic diabetic mouse hind limbs may increase BAG3 expression not only in skeletal muscles but also in other cell types in the treated limbs. Therefore, the improved perfusion recovery observed in BAG3-overexpressing limbs could be due to BAG3 overexpression in vascular cells or indirectly through BAG3 supporting healthier skeletal muscles, which in turn supports better vascular adaptation to the ischemic injury. Previously, Falco et al. demonstrated that BAG3 is expressed in endothelial cells, where its absence leads to decreased angiogenesis. They demonstrated that BAG3 removal in endothelial cells resulted in reduced binding of dual-specificity phosphatase 6 (DUSP6) to extracellular signal-regulated kinase (ERK), which results in sustained phosphorylation of ERK, leading to cell cycle arrest [39]. Lastly, our immunostaining results showed evidence of increased muscle regeneration and less severe muscle injury in ischemic diabetic GAs with BAG3 overexpression. Taken together, this suggests the level of BAG3 expression is critical to protect the skeletal muscles from ischemic injury, and BAG3 insufficiency may be a key pathologic process in diabetic PAD.

In the present study, we have demonstrated that simulated ischemia enhanced BAG3 expression in human skeletal muscle cells. Our findings are consistent with that of other studies, which showed increased BAG3 expression in cardiomyocyte and tumor cell lines exposed to hypoxia [40,41]. Lower BAG3 expression in ischemic human skeletal muscle cells is associated with decreased cell viability in vitro. This is also consistent with prior studies showing decreased cardiomyocyte viability following siRNA-mediated knockdown of BAG3 [40]. In vivo, lower BAG3 expression in ischemic diabetic mouse skeletal muscles is associated with decreased autophagy and increased necroptosis. In contrast, in vivo, overexpression of BAG3 in ischemic diabetic skeletal muscles enhances autophagy, decreases necroptosis, muscle injury, and limb necrosis and enhances muscle regeneration and function. Our findings point to BAG3 as a possible therapeutic target to overcome the severe post-ischemic skeletal muscle injury associated with CLTI in diabetics with PAD.

## 4. Materials and Methods

### 4.1. Animals

All the experiments involving animals were approved by the Institutional Animal Care and Use Committee (IACUC) of the University of Iowa, Iowa, in accordance with the NIH guide for the care and use of laboratory animals. All experiments were conducted with male C57BL/6J mice (Cat# 000,664 Jackson Laboratory, Bar Harbor, MA, USA). Mice around 10–12 weeks were fed a normal chow diet (NCD, Cat#7913, ENVIGO, Madison, WI, USA) or a high-fat diet (HFD, 60.3% kcal, Cat#TD.06414, ENVIGO, Madison, WI, USA) with free access to food and water for 16 weeks and were used around 26–28 weeks of age for the experiments. Glucose tolerance was assessed by intraperitoneal glucose tolerance test (IPGTT) in unanaesthetised mice [13]. Briefly, 1 mg/g mouse of glucose was administered intraperitoneally, and blood glucose from the tail vein was measured at 0, 15 min, 30 min, 1 and 2 h. The area under the curve (AUC) was adjusted by the weight of the animal for comparison between the groups. Based on the AUC of NCD-fed mice (NCD AUC between 379 and 567), an AUC > 634 was considered to be impaired glucose tolerance [13]. Only HFD mice meeting these parameters were used in the experiment. IPGTT was performed prior to randomization of animals into the experimental groups. Experimental HFD groups were randomized into 2 treatment groups that received AAV6-EV (control mice with empty vector) or AAV6-BAG3 (BAG3 overexpression). Mice were euthanized by CO2 exposure followed by cervical dislocation to assure euthanasia.

### 4.2. In Vivo Adenoviral Injection

AAV6 encoding an empty vector alone or AAV6 with the vector plus mouse BAG3 cDNA expression under the CMV promoter (1 × 10^11^ per limb) was injected into the gastrocnemius and tibialis anterior muscles of anesthetized C57BL/6J mice fed with HFD at 5 days and 1 day prior to HLI surgery.

### 4.3. Cell Culture and Simulated Ischemia

Commercially available cultures of human skeletal muscle cells were used in the study. For the non-ischemic condition, human skeletal muscle cells (HSMCs; Cat#150p-05a; Cell Applications, Inc., San Diego, CA, USA) were grown in human skeletal muscle cell culture media (Cat#151-500; Cell Applications, Inc., San Diego, CA, USA) containing 20% FBS (Cat#10437-028; Thermo Fisher Scientific, Waltham, MA, USA) at 37 °C in a humidified 95% air and 5% CO_2_ atmosphere. All experiments were done with HSMCs grown between passages 4 and 10. For simulated ischemia, cells were cultured in starvation medium (Cat#151S-250; Cell Applications, Inc., San Diego, CA, USA) and hypoxia (2% oxygen, 5% CO_2_) using a hypoxic chamber at 37 °C for the indicated time [12,20].

### 4.4. In Vitro Plasmid Transfection

HSMCs were transfected either with plasmids coding shRNA Control (shControl, TR30013, Origene, Rockville, MD, USA) or shRNA to BAG3 (shBAG3, Cat#TRCN0000292298, Sigma-Aldrich, St. Louis, MO, USA) using Lipofectamine 3000 transfection reagent (Cat#L3000-015; Thermo Fisher Scientific, Waltham, MA, USA) according to the manufacturer’s instructions. After 48 h of transfection, cells were used for experiments.

### 4.5. Cell Morphology and Viability Assay

HSMCs transfected with shControl or shBAG3 were exposed to simulated ischemia for 0, 24, or 48 h and imaged with a digital camera (MD35A, AmScope, Irvine, CA, USA) attached to the microscope. At the indicated time, cells were trypsinized and stained with trypan blue (0.4%), and viable cells were counted using a hemocytometer. The results were presented as percentage (%) cell viability compared to non-ischemic cells.

### 4.6. TaqMan qPCR

Total RNA was isolated from the cells or muscle tissue using the RNeasy Mini Kit (Qiagen, Germantown, MD, USA). RNA concentrations were determined using Nanodrop 2000 (Thermo Fisher Scientific, Waltham, MA, USA). One µg of total RNA for each sample was reverse-transcribed into cDNA using the High-capacity RNA to cDNA synthesis kit (Cat#4388950; Thermo Fisher Scientific, Waltham, MA, USA) and was diluted to 100 µL with sterile water. Equal amounts (45 ng per reaction) of cDNA were used for a gene-specific TaqMan PCR for BAG3 expression (TaqMan Fast Advanced Master Mix, Cat#4444554, Thermo Fisher Scientific, Waltham, MA, USA) in a Quant Studio 3 thermocycler (Applied Biosystems, Thermo Fisher Scientific, Waltham, MA, USA). The results were analysed by the ∆∆Ct method [11,12,20] and presented as fold change vs. control. Ct values of >35 were considered as no expression of the gene.

### 4.7. HLI Surgery, Perfusion Recovery and Limb Necrosis Score

HLI surgery was performed as described previously [11,12,20]. Briefly, the mice were anaesthetized by intraperitoneal injection of xylazine (5 mg/kg) and ketamine (100 mg/kg) followed by surgical ligation and excision of the femoral artery of the left hind limb. The unoperated right hind limb served as the non-ischemic control. The peripheral blood flow was measured by laser speckle contrast imaging using Perica PSI (Perimed, Las Vegas, NV, USA). Blood perfusion images were obtained on days 0 (immediately after surgery), 3, 7, 14 and 21 post-surgery. The perfusion recovery in the hind limbs was expressed as the ratio of the operated to the contra-lateral hind limb blood flow using the manufacturer’s software. Necrosis score in post-surgery limbs was measured as described previously [21,42]. Briefly, necrosis was scored as follows: Stage 1, involving only toes; Stage 2, extending to the dorsum pedis; Stage 3, extending to the crus; and Stage 4, extending to the ankle or complete loss of the foot.

### 4.8. Western Blots

HSMC lysates or hindlimb muscle tissue (gastrocnemius) homogenates were prepared in RIPA buffer (Cat#89901; Pierce RIPA Buffer, Thermo Fisher Scientific, Waltham, MA, USA) with 0.5M EDTA and protease/phosphatase inhibitors. Day 3 post-HLI was chosen for protein and mRNA assessment because we previously showed there was no difference in perfusion between HFD and NCD mice [13]. Equal amounts of protein were separated on 4–12% Bis-Tris Gel (Cat#NW04127BOX; Thermo Fisher Scientific, Waltham, MA, USA) and transferred to a nitrocellulose membrane. Following incubation in blocking buffer (Cat#927-40000, Odyssey blocking buffer, LI-COR, Lincoln, NE, USA) for 60 min, membranes were incubated overnight at 4 °C in primary antibodies (1:1000 dilution in blocking buffer) against BAG3 (Cat#NBP227398, Centennial, CO, USA), LC3A/B (Cat#12741, Cell Signaling Technology, Danvers, MA, USA), ATG5 (Cat#12994, Cell Signaling Technology, Danvers, MA, USA), ATG7 (Cat#8558, Cell Signaling Technology, Danvers, MA, USA), RIP3 (Cat#PA519956, Invitrogen, Waltham, MA, USA), RIP1 (Cat#3493, Cell Signaling Technology, Danvers, MA, USA), and MLKL (Cat#PA5-43960, Invitrogen, Waltham, MA, USA). The membrane was washed with Tris-buffered saline with 0.1% Tween20 (TBST) and incubated with secondary antibodies (1 in 5000 dilutions; IRDye 800CW Goat anti-Rabbit; Cat#926-32211; LI-COR, Lincoln, NE, USA) for 1 h at room temperature, followed by TBST wash. Signals were captured by an iBright Fl1500 Imaging System (Thermo Fisher Scientific, Waltham, MA, USA). Prior to probing with antibodies, all membranes were stained with Ponceau S solution and imaged.

### 4.9. Western Blots Quantification

The signal intensity of protein bands on images of Western blots and Ponceau S-stained membranes was measured using Image Studio Lite Ver 5.2 software (Software version 5.2, LI-COR, Lincoln, NE, USA). Target protein band signals were normalized to the total protein present in the corresponding lanes (Ponceau S staining of membrane), and the ratio of band intensity to total protein staining was presented as fold change versus controls. The representative blot of Ponceau S staining is presented in the results. Wherever more than one Western blot is shown in the same figure, the total protein staining for other proteins is presented in the Supplementary Figures S1–S3. We have used Ponceau S-stained total protein for normalization since we have found that expression of commonly used housekeeping genes (GAPDH, β-actin, α-tubulin) is highly variable between normoxic and ischemic conditions. Moreover, Ponceau S staining has been shown to be more reliable as a measure of protein loading than many housekeeping genes [13,43].

### 4.10. Muscle Contractile Function

The maximal isometric torque of the ankle gastrocnemius muscle (GA) was measured in control and ischemic limbs using the 1300A 3-in-1 Whole Animal System (Aurora Scientific, Aurora, ON, Canada) as previously described [44]. Animals were kept under anesthesia (3% isoflurane via a nose cone) throughout the measurement process. The foot was immobilized with adhesive tape to a footplate of a torque cell. Resting tension and muscle length were continually adjusted for each muscle to obtain the optimal twitch contraction force. A subcutaneous electrode was used to stimulate the ankle dorsiflexors via the fibular nerve. Proper electrode position was determined by a series of isometric twitches. After a 5-min equilibration period, isometric tension was evaluated with stimulations of 150 Hz for 300 ms. Data were analyzed to determine muscle torque using the Dynamic Muscle Analysis software (ASI 611A v.5.321; Aurora Scientific).

### 4.11. Immunohistochemistry

Gastrocnemius muscle tissue samples were fixed in 10% Formalin, as described earlier [11,12,20]. Briefly, 10 µM thick sections of ischemic hindlimb muscle (GA) were subjected to H & E staining for muscle morphology analysis. The extent of tissue injury was expressed as the total number of muscle fibers with centrally located nuclei to the total number of muscle fibers counted [13,45]. Capillaries were identified using a rat anti-mouse CD31 antibody (1 in 1000 dilution; Cat#NB100-64796, Novus Biologicals, Littleton, CO, USA), and muscle regeneration was assessed by using a mouse anti-human eMyHC antibody (1 in 1000 dilution; Cat#F1.652, DSHB, University of Iowa, Iowa, IA, USA). All slides were scanned at 20× magnification under a fluorescence microscope (BX61, Olympus corporation, Tokyo, Japan). Stained cells and muscle fibers in the entire sections were counted, capillary density was expressed as CD31+/myofiber, and muscle regeneration was expressed as a percentage of eMyHC+ myofibers.

### 4.12. Statistical Analysis

All data are presented as mean ± SEM. Statistical comparison between groups was analysed by one-way ANOVA using Tukey’s posthoc test by GraphPad Prism, and a *p*-value < 0.05 was considered statistically significant.

## Data Availability

Not applicable.

## Supplementary Materials

The supporting information can be downloaded at: https://www.mdpi.com/article/10.3390/ijms231810715/s1.
