# Supplementary material for: BAG3 Attenuates Ischemia-Induced Skeletal Muscle Necroptosis in Diabetic Experimental Peripheral Artery Disease
Source: Int J Mol Sci. 2022 Sep 14;23(18):10715. doi: 10.3390/ijms231810715 (PMC9502689; doi:10.3390/ijms231810715)

Supplementary Figure S1

S1A: Ponceau S staining of RIP3 (Figure 3C)

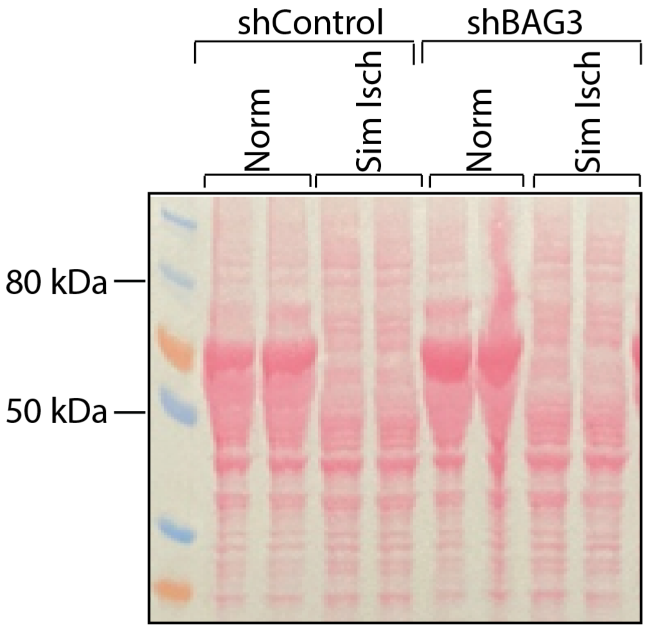

S1B: Ponceau S staining of LC3 (Figure 3C)

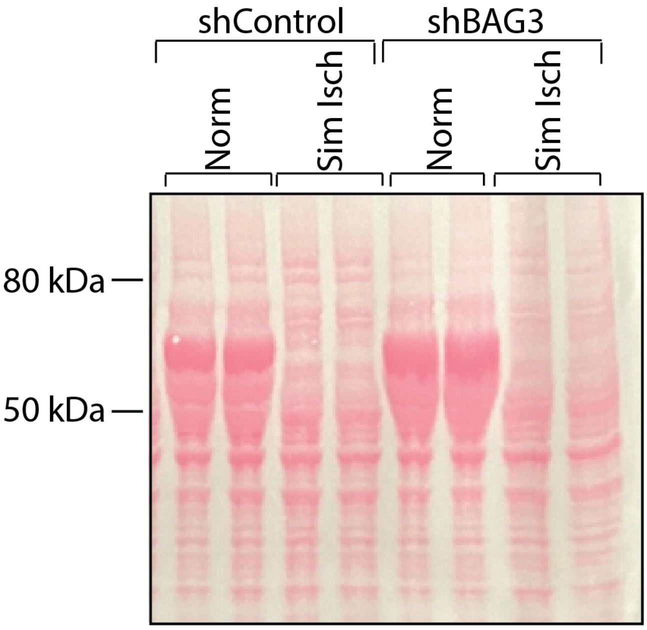

Supplementary Figure S2

S2-A: Ponceau S staining of ATG5 (Figure 4A)

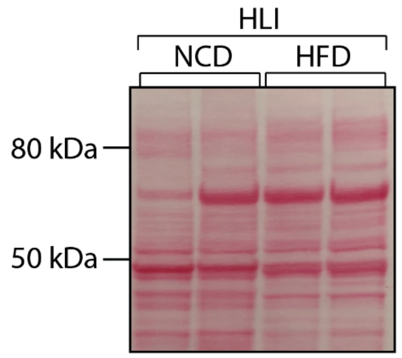

S2-B: Ponceau S staining of ATG7 (Figure 4A)

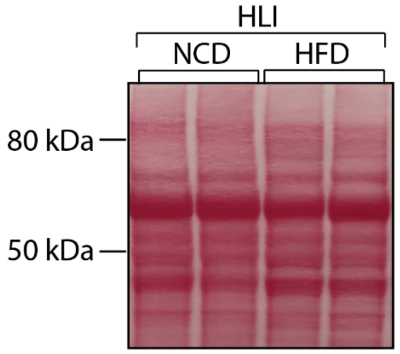

S2-C: Ponceau S staining of LC3 (Figure 4A)

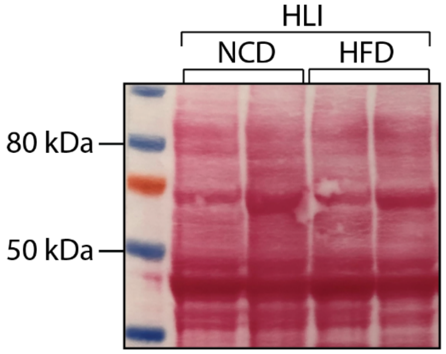

S2-D: Ponceau S staining of RIP3 (Figure 4A)

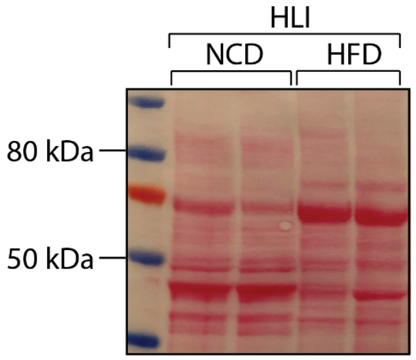

S2-E: Ponceau S staining of RIP1 (Figure 4A)

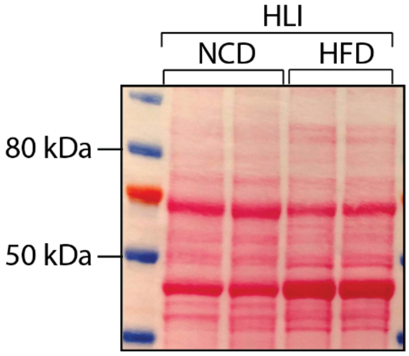

S2-F: Ponceau S staining of MLKL (Figure 4A)

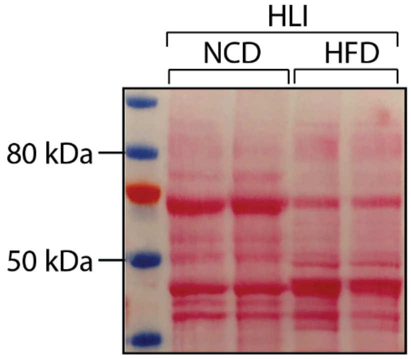

Supplementary Figure S3

S3-A: Ponceau S staining of ATG5 (Figure 5A)

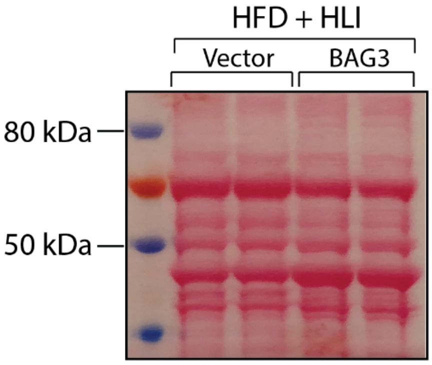

S3-B: Ponceau S staining of ATG7 (Figure 5A)

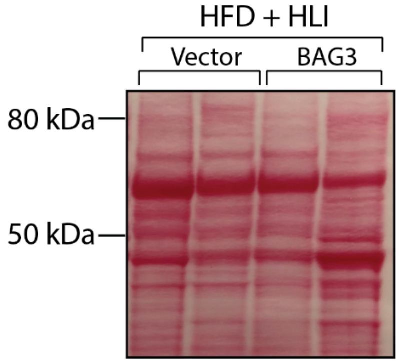

S3-C: Ponceau S staining of LC3 (Figure 5A)

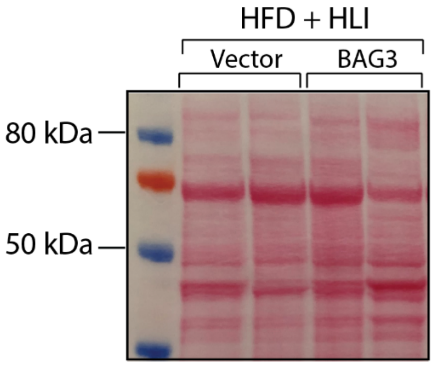

S3-D: Ponceau S staining of RIP3 (Figure 5A)

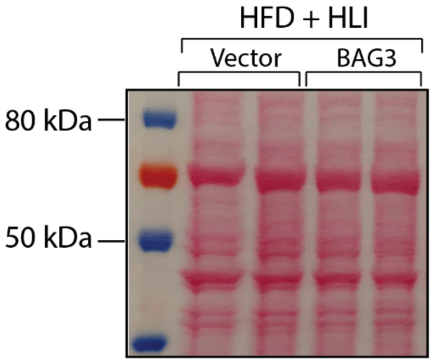

S3-E: Ponceau S staining of RIP1 (Figure 5A)

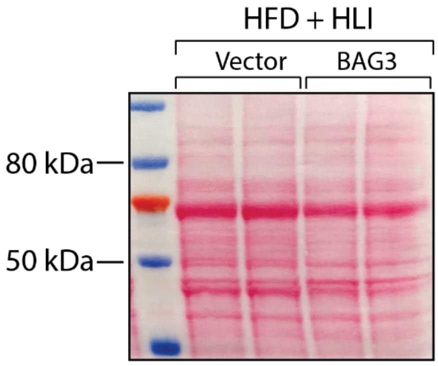

S3-F: Ponceau S staining of MLKL (Figure 5A)

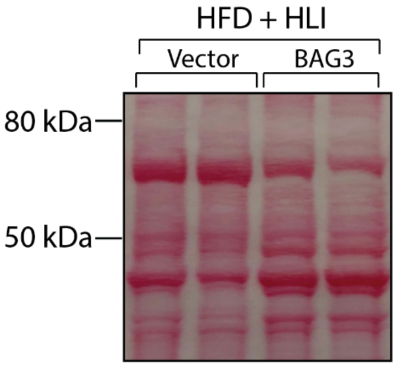

Supplement: Supplementary file 1 [file ijms-23-10715-s001.zip › ijms-1899558-supplementary.pdf]
